# Immune reconstitution of HLA-A*0201/BMLF1- and HLA-A*1101/LMP2-specific Epstein Barr virus cytotoxic T lymphocytes within 90 days after haploidentical hematopoietic stem cell transplantation

**DOI:** 10.1186/s12985-019-1123-y

**Published:** 2019-02-08

**Authors:** Ling Zhou, Dao-pei Lu

**Affiliations:** 1 The Fifth People’s Hospital of Shanghai, Fudan University, No. 801 Heqing Road, Minhang County, Shanghai, 200240 China; 2 Shanghai Dao-Pei Hospital, No. 126 Ruili Road, Minhang County, Shanghai, 200240 China

**Keywords:** Haploidentical hematopoietic stem cell transplantation, Epstein-Barr virus, CD8 cells, Pentamers

## Abstract

**Background:**

Haploidentical hematopoietic stem cell transplant (haplo-HSCT) recipients are at high risk for Epstein Barr virus (EBV)-related diseases. EBV-specific CD8^+^ cytotoxic T cells can control EBV-infected B cell expansion; however, no studies have investigated EBV-specific immune reconstitution after HSCT, particularly haplo-HSCT. Therefore, in this study, we aimed to characterize EBV-specific immune cell reconstitution after haplo-HSCT.

**Methods:**

HLA-A*1101 and HLA-A*0201 pentamers folded with immunodominant EBV peptides were used to detect EBV-specific CD8^+^ T cells by flow cytometry in peripheral blood mononuclear cells from 19 haplo-HSCT recipients and the results were compared with those in controls. We also compared the EBV-specific pentamer-binding cell frequencies in patients with or without EBV-related diseases by flow cytometry.

**Results:**

Pentamer-binding EBV-specific CD8^+^ T cells were detected at + 30, + 60 and + 90 days after haplo-HSCT in EBV-seropositive patients subjected to haplo-HSCT from an EBV-seropositive donor. The frequencies of the HLA-A*0201/BMLF1-GLC pentamer in haplo-HSCT patients at + 30 days were significantly lower than those in HLA-A*0201-positive healthy controls (*p* = 0.019) and patients at + 60 days (*p* = 0.003). The frequencies of the HLA-A*1101/LMP2-SSC pentamer at + 30, + 60, and + 90 days were significantly decreased compared with those in healthy controls (*p* = 0.009, 0.019, and 0.039, respectively); however, the frequencies of the HLA-A*1101/LMP2-SSC pentamer did not differ significantly among patients at + 30, + 60, and + 90 days (*p* = 0.886). There was a significant difference in the frequency of the HLA-A*0201/BMLF1-GLC pentamer at + 60 days between patients with and without EBV-related diseases (*p* = 0.024). Patients with EBV-related diseases showed lower percentages of HLA-A*0201/BMLF1-GLC specific CD8^+^ T cells.

**Conclusions:**

Haplo-HSCT recipients could generate EBV-specific CD8^+^ T cells within + 30 days after transplantation. The HLA-A*0201/BMLF1-GLC pentamer cell frequency at + 60 days may be a useful indicator for monitoring EBV-related diseases in patients after haplo-HSCT. Transfusion with EBV-CTLs within 60 days after haplo-HSCT may have prophylactic effects against EBV-related diseases.

## Background

Epstein-Barr virus (EBV) reactivation frequently occurs after hematopoietic stem cell transplantation (HSCT) [1, 2]; however, EBV-related diseases, including EBV-related post-transplant lymphoproliferative disorders (PTLDs), can cause significant morbidity and mortality in HSCT recipients. Before the year 2000, mortality from PTLDs after HSCT reached 84.6% [3]. Considerable improvements in outcomes have been achieved since the introduction of new approaches for EBV-related diseases/PTLDs, including monitoring of EBV-DNA by polymerase chain reaction (PCR), pre-emptive therapy, and timely treatment with rituximab; however, mortality remains high, and approximately one-third of patients diagnosed with EBV-PTLDs die [4].

Factors associated with a high risk of EBV-PTLDs include HLA mismatch, EBV serology donor/recipient mismatch (donor+/recipient–), grade II–IV acute graft-versus-host disease (aGVHD), pre-transplant splenectomy, and treatment with mesenchymal stem cells [5]. Furthermore, a high incidence of PTLDs has been reported in pediatric patients who have undergone reduced intensity conditioning (RIC) regimens including antithymocyte globulin (ATG) or alemtuzumab (Campath) [6, 7]. Thus, compared with other types of HSCT recipients, those undergoing haploidentical HSCT (haplo-HSCT) may be at a higher risk of EBV-related diseases, including PTLDs. HLA disparity and ATG use are important components of EBV reactivation after haplo-HSCT.

The primary aim of treatment in patients with EBV-related diseases/PTLDs is to recover EBV-specific immunity [8], as indicated by numerous clinical trials using EBV-specific cytotoxic T lymphocytes (EBV-CTLs) derived from stem cell donors or third-party banks [9–11]; however, little is known about EBV-specific immune reconstitution after HSCT, particularly in haplo-HSCT recipients. Thus, there is no generally accepted time point for transfusion of EBV-CTLs as prophylaxis against the occurrence of EBV-related diseases/PTLDs during the initial period after HSCT.

In this study, we used class I major histocompatibility complex pentamers, HLA-A*0201/BMLF1-GLC and HLA-A*1101/LMP2-SSC, to investigate the dynamics of EBV-CTL immune reconstitution in 19 patients who underwent haplo-HSCT.

## Methods and materials

### Patients and collection of blood samples

Twenty-eight patients undergoing haplo-HSCT using RIC regimens at Shanghai Dao-pei Hospital between November 2012 and January 2015 were prospectively included in this study.

All patients underwent single haplo-HSCT from donors who were HLA-A*0201- or HLA-A*1101-positive. Nine patients were excluded because they relapsed (three cases) or were treated with donor lymphocyte infusion (six cases) within 100 days after transplantation; finally, 19 evaluable patients were enrolled in this study.

The donors for the 19 patients enrolled were positive for HLA-A*0201 (10 cases) or HLA-A*1101 (9 cases), the 28 healthy volunteers (all of them were long-term EBV carriers) carried HLA-A*0201 (9 cases) or HLA-A*1101 (19 cases), and the 49 negative controls were both HLA-A*0201- and HLA-A*1101-negative. Patients were divided into HLA-A*0201 and HLA-A*1101 groups according to the tissue type of their donors. The patients, their donors, healthy long-term EBV carriers, and negative controls all showed negative reactions in an EBV-IgM serum array and positive reactions in an EBV-IgG serum array. Allpatients were 100% donor types by bone punctures and chimerism examinations at + 30, + 60, and + 90 days after transplantation during their lifetimes.

Peripheral blood samples (5 mL) were collected from patients during routine laboratory blood draws. Blood samples from 28 healthy volunteers who were HLA-A*0201- or HLA-A*1101-positive were obtained to identify the baseline level of EBV-CTLs in healthy long-term EBV carriers. Blood samples from HLA-A*0201- and HLA-A*1101-negative volunteers were obtained and used as negative controls. Forty-eight blood samples were obtained from the 19 patients. In the HLA-A*0201 group, blood samples were obtained from five patients gave blood samples at + 30, + 60, and + 90 days; from four patients at + 30 and + 60 days; and from one patient at + 30 days after haplo-HSCT. One patient died at + 43 days because of invasive pulmonary aspergillosis, two patients died at + 74 and + 87 days after haplo-HSCT because of EBV-related diseases/PTLDs, and two patients were lost to follow-up at + 90 days (they did not die, and returned to follow-up at + 120 days). We found that the patient who died at + 43 days was a 100% donor type by bone puncture and short tandem repeat (STR) examination at + 30 days after transplantation. However, at + 33 days, this patient developed hyperthermia, pulmonary infection, and decreased blood cells and was found to have decreased chimerism to 23% of that of the donor recipient at + 37 days. Secondary graft failure was observed, which was thought to be related to the serious pulmonary fungal infection. In the HLA-A*1101 group, blood samples were obtained from six patients at + 30, + 60, and + 90 days and from three patients at + 30 and + 60 days; the other three patients died because of invasive pulmonary aspergillosis (one case), cytomegalovirus (CMV) pneumonia (one case), and EBV-related gastroenteritis (one case).

### Assay to detect serum EBV-specific antibody status

The serum EBV-IgM and IgG statuses before HSCT in patients, their donors, the healthy long-term EBV carriers, and negative controls were detected using an anti-Epstein Barr virus (EBV-VCA) IgM human in vitro Enzyme-Linked Immunosorbent Assay (ELISA) kit and an EBV-VCA-IgG human in vitro ELISA kit (EK-Bioscience, Shanghai, China) according to the manufacturer’s instructions.

### HLA disparity

HLA-A, HLA-B, HLA-Cw, and HLA-DRB1 typing was performed using high-resolution DNA techniques according to the manufacturer’s instructions. The reagents (Special Monoclonal Tray-Asian HLA Class I and Micro SSP HLA Class I and II ABDR DNA Typing Tray; One Lambda, Canoga Park, CA) were commercially imported.

### Chimerism analyses

Bone marrow chimerism was detected every month for six months after haplo-HSCT. Chimerism was determined by one of two methods: DNA fingerprinting of STR, and chromosomal fluorescence in situ hybridization (FISH). Chimerism was evaluated by DNA fingerprinting of STR in recipient BM cells in sex-matched donor-recipient pairs; however, in sex-mismatched donor-recipient pairs, chimerism was analyzed by FISH.

### Haplo-HSCT process

Conditioning regimens were as follows: intravenous administration of cytarabine (2.0 g/m^2^) once daily on days − 13 to − 12, fludarabine (30 mg/m^2^) once daily on days − 11 to − 7, busulfan (0.8 mg/kg, every 6 h) on days − 6 to − 4, and ATG (Fresenius, 5 mg/kg) once daily on days − 5 to − 2 (11 patients); or intravenous administration of cytarabine (2.0 g/m^2^) once daily on days − 14 to − 13, fludarabine (30 mg/m^2^) once daily on days − 12 to − 8, thiotepa (125 mg/m^2^) once daily on days − 8 to − 5, and ATG (Fresenius, 5 mg/kg) once daily on days − 5 to − 2 (eight patients).

For GVHD prophylaxis and management, each patient was administered cyclosporine A (CSA, 2.5 mg/kg twice daily, intravenously), a short course of methotrexate (15 mg/m^2^ once daily, intravenously, on day + 1 and 10 mg/m^2^ on days + 3 to + 5), and oral mycophenolate mofetil (7.5 mg/kg twice daily) from days + 1 to + 14 as prophylaxis for GVHD. CSA was withdrawn if the patients showed viral reactivation, including CMV or EBV, and did not have GVHD at that time. Methylprednisolone (0.25 mg/kg/day) was administered if patients developed aGVHD < grade II. Only two patients suffered from grade III aGVHD, which occurred before CSA was tapered.

Collection of hematopoietic stem cells was performed as follows: donor marrow and peripheral blood mobilized using granulocyte-colony stimulating factor (7.5 μg/kg/day, on day − 4 to day 02) were harvested on day 01 and 02, respectively.

Supportive care was similar for all patients. All blood products were irradiated and leukocyte-depleted. Antifungal prophylaxis was routinely administered with caspofungin during the conditioning process and voriconazole was administered after transplantation. Pneumocystis prophylaxis, typically trimethoprim-sulfamethoxazole, was administered until 6 months post-transplantion. Acyclovir, typically 400 mg orally twice per day, was administered until 24 months post-transplantation.

### Monitoring of EBV reactivation in peripheral blood and definition of EBV-related diseases

The EBV-DNA copy number was determined by quantitative PCR (qPCR) using peripheral blood samples or biopsy specimens. Briefly, genomic DNA was isolated from 250 μL whole blood or digestive tissues with an Axyprep Mag tissue-blood genomic DNA kit (Axygen, Union City, CA,USA) according to the manufacturer’s instructions. qPCR was performed on an ABI 7300 thermal cycler using commercially available PCR kits for EBV (Daan Gene Technology, Guangzhou, China) and the results were shown as DNA copies/mL of whole blood, with a detection threshold of 500 copies/mL for EBV.

EBV-DNA copy numbers were monitored weekly during the first 30 days after haplo-HSCT and subsequently every other week until 3 months after transplantation or until EBV-DNA became undetectable in the peripheral blood.

EBV-DNAemia was defined as EBV-DNA loads of more than 500 copies/mL at two consecutive time points, without any signs or symptoms of EBV-related diseases, including probable and proven PTLDs. Probable PTLDs were defined as significant lymphadenopathy, hepatosplenomegaly, or other end-organ manifestations, accompanied by a high EBV-DNA blood load and the absence of tissue biopsy and other documented causes [12]. Proven PTLDs were diagnosed as detection of EBV nucleic acids or EBV-encoded proteins in a tissue specimen, together with symptoms and/or signs from the affected organ [12].

### MHC class I-peptide pentameric complexes (pentamers) and reagents

Two EBV peptide epitopes were used: the HLA-A*0201-restricted epitope, GLCTLVAML, derived from the lytic cycle protein BMLF1 (amino acids 259–267), and the HLA-A*1101-restricted epitope SSCSSCPLSK, derived from the latent cycle protein, LMP2 (amino acids 340–349). The pentamers, HLA-A*0201/BMLF1-GLC and HLA-A*1101/LMP2-SSC, were purchased from ProImmune, Ltd. (Oxford, UK). Pentamers were labeled with phycoerythrin (PE). Monoclonal antibodies, including anti-CD3 (peridinin chlorophyll protein-[PerCP]), anti-CD8 (fluorescein isothiocyanate-[FITC]), and anti-CD19 (allophycocyanin-[APC]), were purchased from BD Biosciences (Franklin Lakes, NJ, USA). The experimental protocol and operation were recommended by the Pentamer Handbook from ProImmune, Ltd. (Oxford, UK). According to the protocol that cells obtained from a mismatched pentamer (irrelevant MHC allele and/or irrelevant peptide). can be used to control non-specific staining, and that exclusion of B cells is likely to reduce most of the non-specific background, we used both HLA-0201- and 1101-negative adult volunteers as negative controls and added anti-CD19 monoclonal antibody to remove B lymphocytes. Briefly, forward and side scatters were used to gate viable populations of cells, and CD3^+^CD19^−^ cells were then gained to delete B lymphocytes. Next, CD3^+^CD19^−^CD8^+^ cells were gained as CD8^+^ T lymphocytes, and finally, pentamer-stained CD3^+^CD8^+^CD19^−^ cells were gained as target cells for FACS analysis. The percentage of EBV-CTLs was calculated as follows: percentage EBV-CTLs = (percentage EBV-CTLs in patients or percentage EBV-CTLs in healthy long-term EBV carriers (healthy controls) – percentage EBV-CTLs in negative controls) × 100%.

Blood samples were collected from patients at + 30, + 60, and + 90 days after haplo-HSCT, whereas blood samples were collected only once from healthy volunteers as HLA-A*0201- or HLA-A*1101-positive and negative controls. We evaluated the performance of HLA-A*0201/BMLF1-GLC and HLA-A*1101/LMP2-SSC pentamers based on the percentages of EBV-specific CD8^+^ T cells by four-color flow cytometry. All experiments were performed in triplicate.

### Pentamer staining assays

FACS lysing solution (BD Biosciences) was added to the blood samples to lyse the erythrocytes. Cells were washed twice and resuspended in phosphate-buffered saline (PBS) containing 1% fetal calf serum (FCS) and 0.1% sodium azide. The number of nucleated cells was counted under a microscope and adjusted such that each tube contained 1–2 × 10^6^ nucleated cells. Titrated pentamers were then added to the samples. After incubation for 10 min at room temperature (25 °C) without light, titrated monoclonal antibodies (PerCP-, FITC-, or APC-conjugated CD3, CD8, or CD19, respectively) were added for surface staining. After incubation for 20 min at 4 °C without light, the cells were washed twice with PBS containing 1% FCS and 0.1% sodium azide and then stored in 1% paraformaldehyde at 4 °C. Samples were analyzed on a FACS Calibur instrument (BD Biosciences) within 6 h. At least 5000 live CD3^+^ lymphocytes were counted by flow cytometry. CellQuest (BD Biosciences) was used for analysis. The frequencies of pentamer-binding CD8^+^ T cells were calculated based on the percentages of CD3^+^CD8^+^CD19^**−**^ T cells that bound to the pentamers. Blood samples from HLA-A*0201- and HLA-A*1101-negative patients were used as negative controls.

### Statistics

Statistical analysis was performed using SPSS 21.0 software (SPSS, Inc., Chicago, IL, USA). Quantitative data with normal distributions were expressed as the mean ± standard deviation. Continuous variables and quantitative data without normal distributions were expressed as the median and range. The percentages of pentamers between two groups were compared using independent sample *t* tests. Wilcoxon rank-sum tests were used to compare the frequencies of HLA-A*0201/BMLF1-GLC pentamers at + 60 days between patients with and without EBV-related diseases. Analysis of variance was used to compare the percentages of pentamer-reactive cells among multiple groups. All analyses were two-tailed and *p* < 0.05 was considered significant.

## Results

### Patient characteristics

The characteristics of the 19 patients included in this study are presented in Table 1. The general data including sex (*p* = 0.770), age (*p = 0.967*), diseases (*p* = 0.669), and conditioning regimens (*p* = 0.849) were not significantly different between the HLA-A*0201 and 1101 groups.

**Table 1:** Patient characteristics

Characteristics	Cases (*n* = 19)
Patient age, median (range), years	33 (3–57)
Sex	
Male	13
Female	6
Diagnosis	
AML	3
ALL	7
MDS	7
PID	2
Pre-HSCT EBV status, donor/recipient	
Positive/positive	19
MNC, median (range), × 10^8^/kg	5.17 (2.53–9.59)
CD34+ cell absolute count, median(range), ×10^6^/kg	6.37 (1.61–11.13)
CD3+ cell absolute count, median(range), ×10^8^/kg	2.76 (0.82–7.83)
Engraftment	
White cells, median (range), days	12 (10–18)
Platelets, median (range), days	13 (9–18)
Acute graft-versus-host disease, n	
No	7
I–II degree	10
III–IV degree	2
EBV infection after HSCT within 100 days	
No	9
EBV-DNAemia	5
EBV disease	5
CSA stop	
No	4
Yes	15
CSA stopping time, median (range), days	32.5 (4–88)

All the 19 patients showed 100% chimerism at + 30 days after haplo-HSCT. One patient in the HLA-A*0201 group lost the 100% chimerism at + 37 days after HSCT because of severe invasive pulmonary aspergillosis and died at + 43 days. The other patients sustained the 100% chimerism during their lifetimes.

EBV-DNA was detected in blood or tissue samples from 10 patients, including five with EBV-DNAemia and five with EBV-related diseases. All five cases with EBV-related diseases were probable PTLDs based on positron emission tomography-computered tomography (PET-CT) imaging or tissue biopsy combined with EBV-DNA copy number assays by qPCR, and histological examination by in situ hybridization to detect EBV-encoded small RNA transcripts or viral antigens was not performed because of the small sample size. In the five patients with EBV-related diseases, three had fever, tonsillitis, adenopathy, and high peripheral blood EBV-DNA loads; EBV-DNA was not detected in the peripheral blood of two patients. However, EBV-DNA was found in intestinal biopsy specimens obtained by colonoscopy in one of these two patients and in cerebrospinal fluid collected by lumbar puncture in the other one.

### Percentages of HLA-A*0201/EBV-BMLF1-GLC-specific CTLs in healthy controls and patients after haplo-HSCT

**Fig. 1:**
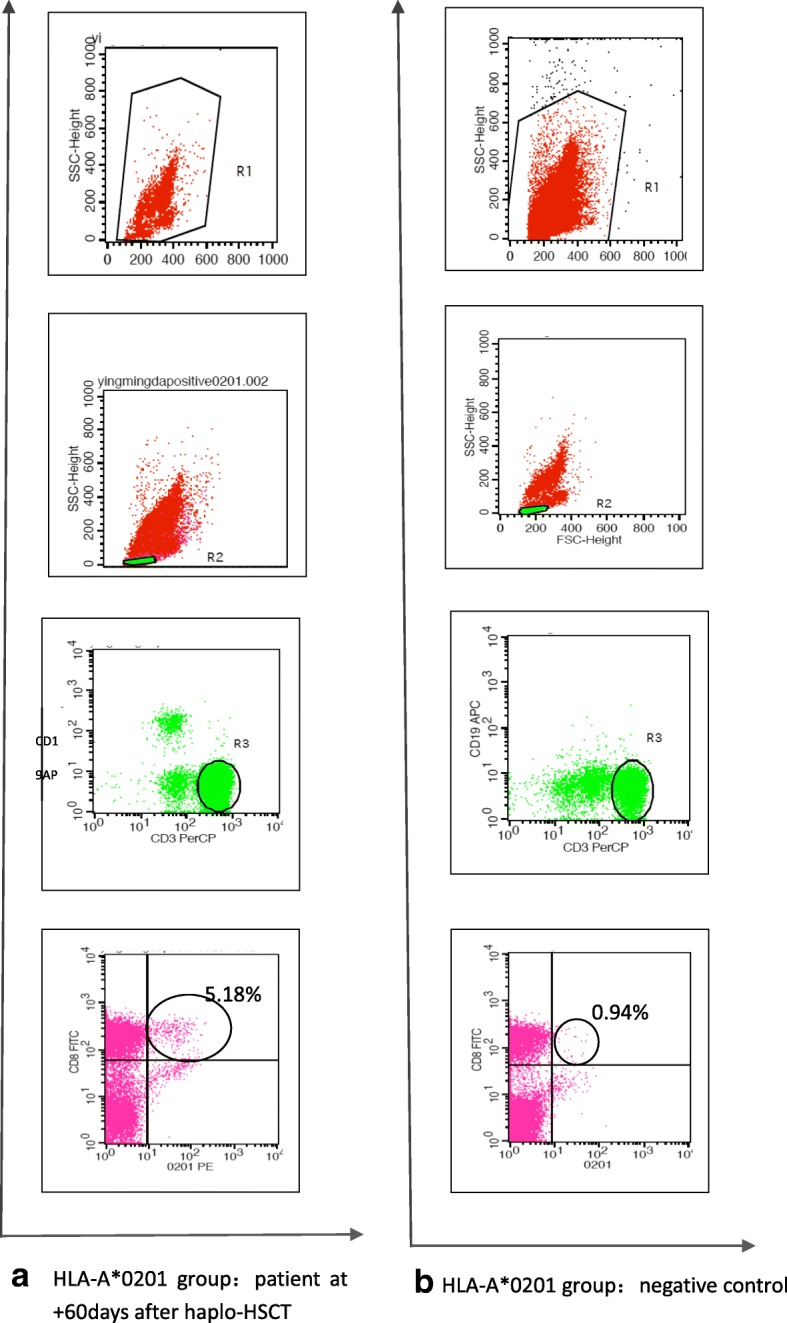
CD8^+^ T cells specific for HLA-A*0201/EBV-BMLF1-GLC peptide by pentamer are present in the peripheral blood of haplo-HSCT recipients. (**a**): PBMCs from an HLA*A0201(+) patient at + 60 days after haplo-HSCT. The four flow graphs show the flow gating and target cell sorting. Forward and side scatters were used to gate viable populations of the cells, and CD3^+^CD19^−^ cells were then gained to delete B lymphocytes. Subsequently, CD3^+^CD19^−^CD8^+^ cells were gained as CD8^+^ lymphocytes, and finally, HLA-A*0201-pentamer stained CD3^+^CD8^+^CD19^−^ cells were gained as the target cells for FACS analysis. **(b)**: PBMCs from HLA*A0201- and HLA*A1101-negative controls were stained with anti-CD3-preCP monoclonal antibodies, anti-CD19-APC monoclonal antibodies, anti-CD8-FITC monoclonal antibodies, and HLA-A*0201/EBV-BMLF1-GLC pentamer-PE, and the cells were analyzed as described in (**a**)

The negative controls were from EBV-seropositive, HLA-A*0201 and 1101-negative patients (Fig. 1).

**Fig. 2:**
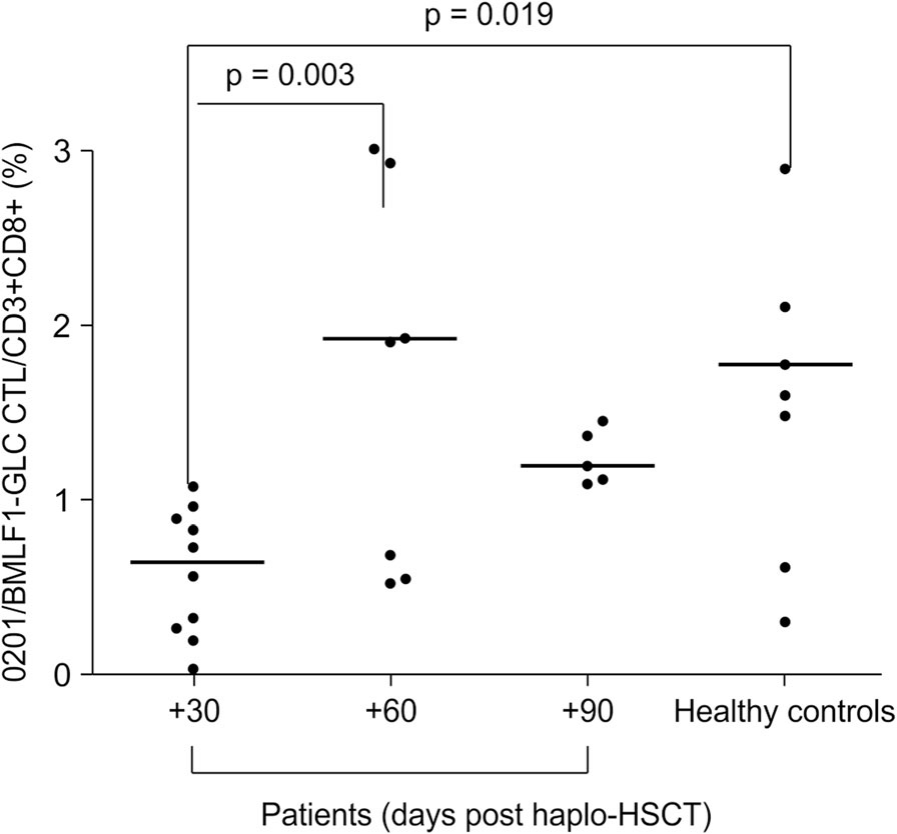
Percentages of HLA-A*0201/EBV-BMLF1-GLC-specific CD8^+^ T cells in patients at + 30, + 60, and + 90 days after haplo-HSCT and in healthy controls. Data are expressed as the means ± standard deviations. The recovery of CD8^+^ T cells against EBV specific epitope BMLF1-GLC as measured by pentamer technology. Four group are shown on the panel. The percentages of HLA-A*0201/EBV-BMLF1-GLC-specific CD8^+^ T cells in patients at + 30, + 60, and + 90 days after haplo-HSCT and in healthy controls are presented

The mean percentages of HLA-A*0201/BMLF1-GLC-specific CTLs were 2.222% ± 1.665% in healthy controls, 0.587% ± 0.365% in patients at + 30 days after haplo-HSCT, 2.101% ± 1.328% in patients at + 60 days post-haplo-HSCT, and 1.244% ± 0.157% in patients at + 90 days after haplo-HSCT. The mean percentage of HLA-A*0201/BMLF1-GLC-specific CTLs at + 30 days was significantly lower than that of HLA-A*0201-positive healthy controls (*p* = 0.019), whereas the mean percentages at + 60 and + 90 days were not significantly different (*p* = 0.867 and 0.117, respectively). A comparison of the three time points (+ 30, + 60, and + 90 days) after haplo-HSCT indicated significant differences between the mean percentages of HLA-A*0201/BMLF1-GLC-specific CTLs at + 30 and + 60 days (*p* = 0.003), whereas the differences between + 30 and + 90 days (*p* = 0.529) and between + 60 and + 90 days (*p* = 0.262) were not significant (Fig. 2).

### Percentages of HLA-A*1101/EBV-LMP2-SSC-specific CTLs in healthy controls and patients after haplo-HSCT

For these experiments, the negative controls also comprised EBV-seropositive, HLA-A*0201- and 1101-negative patients.

**Fig. 3:**
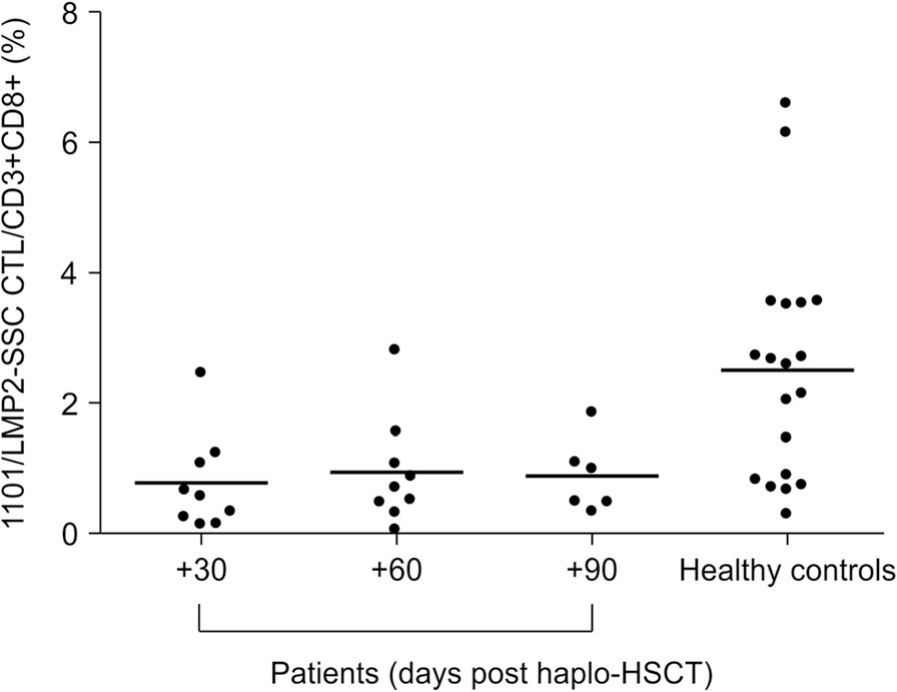
Percentages of HLA-A*1101/EBV-LMP2-SSC-specific CD8^+^ T cells in patients at + 30, + 60, and + 90 days after haplo-HSCT and in healthy controls. Data are presented as the means ± standard deviations. The recovery of CD8^+^ T cells against EBV specific epitope LMP2-SSC as measured by pentamer technology. Four group are shown on the panel. The percentage of HLA-A*1101/EBV-LMP2-SSC-specific CD8^+^ T cells in patients at + 30, + 60, and + 90 days after haplo-HSCT and in healthy controls are presented

The mean percentages of HLA-A*1101/LMP2-SSC-specific CTLs were 2.508% ± 1.766% for healthy controls, and 0.774% ± 0.747% for patients at + 30 days post-haplo-HSCT, 0.945% ± 0.828% for patients at + 60 days post-haplo-HSCT, and 0.886% ± 0.572% for patients at + 90 days post-haplo-HSCT. The mean percentages of HLA-A*1101/LMP2-SSC-specific CTLs at + 30, + 60, and + 90 days were significantly decreased compared with those in healthy controls, (*p* = 0.009, 0.019, and 0.039 respectively). A comparison of the three-time points (+ 30, + 60, and + 90 days) after haplo-HSCT revealed no significant differences among the three groups (*p* = 0.886) (Fig. 3).

### Comparison of HLA-A*0201/EBV-BMLF1-GLC-specific and HLA-A*1101/EBV-LMP2-SSC-specific CTLs in healthy controls and patients after haplo-HSCT

**Fig. 4:**
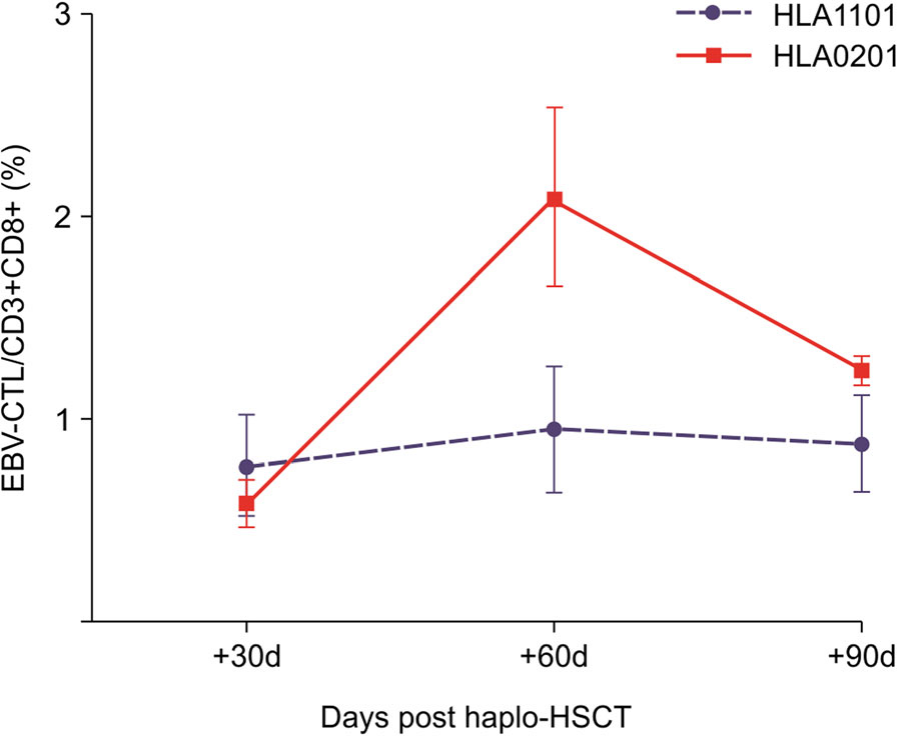
Percentages of HLA-A*0201/EBV-BMLF1-GLC- and HLA-A*1101/EBV-LMP2-SSC-specific CD8^+^ T cells in patients after haplo-HSCT. Data are expressed as the means ± standard deviations. The percentages of HLA-A*1101/LMP2-SSC-specific CTLs and HLA-A*0201/EBV-BMLF1-GLC-specific CTLs in EBV-seropositive healthy controls and in patients at + 30, + 60, and + 90 days after haplo-HSCT are shown. The percentages of HLA-A*1101/LMP2-SSC-specific CTLs and HLA-A*0201/EBV-BMLF1-GLC-specific CTLs in healthy controls were detected at only one-time point. The percentages of HLA-A*1101/LMP2-SSC-specific CTLs in healthy controls were 2.508% ± 1.766%, and the percentages of HLA-A*0201/EBV-BMLF1-GLC-specific CTLs in healthy controls were 2.222% ± 1.665%

The percentages of HLA-A*1101/LMP2-SSC-specific CTLs were similar to those of HLA-A*0201/EBV-BMLF1-GLC-specific CTLs in EBV-seropositive healthy controls (2.508% ± 1.766% vs 2.222% ± 1.665%, *p* = 0.688). The percentages of HLA-A*1101/LMP2-SSC-specific CTLs at + 60 days were significantly lower than those of HLA-A*0201/EBV-BMLF1-GLC-specific CTLs (0.945% ± 0.828% vs 2.101% ± 1.328%, *p* = 0.042). A comparison of the mean percentages of the two EBV-specific CTLs at + 30 and + 90 days revealed no significant differences (0.774% ± 0.747% vs 0.587% ± 0.365 and 0.886% ± 0.572% vs 1.244% ± 0.157%, *p* = 0.491 and 0.211, respectively) (Fig. 4 and Table 2).

**Table 2:** Percentages of EBV specific CTLs in 19 patients at + 30, + 60, and + 90 days after haplo-HSCT and in healthy controls

Groups	Patients/healthy controls (NO.)	Percentages at + 30 days	Percentages at + 60 days	Percentages at + 90 days
HLA-A*0201 group				
	P1	0.1982	2.9100	1.0950
	P2	0.8337	1.9200	1.3644
	P3	0.7296	3.0000	1.0119
	P4	0.3252	3.1700	1.4483
	P5	0.0179	1.9000	1.1990
	P6	1.0790	4.2400	–
	P7	0.5619	0.6900	–
	P8	0.2727	0.5300	–
	P9	0.9600	0.5500	–
	P10	0.8924	**–**	–
	H1	2.1000		
	H2	0.6200		
	H3	1.5900		
	H4	5.7900		
	H5	1.7700		
	H6	0.3000		
	H7	2.8800		
	H8	1.4700		
	H9	3.4800		
HLA-A*1101 group				
	P1	2.4667	0.0672	–
	P2	0.3413	0.5073	0.4926
	P3	0.1585	0.3339	0.3434
	P4	1.2534	1.5635	1.0086
	P5	1.0931	2.8223	1.1053
	P6	0.2601	0.5272	–
	P7	0.6569	0.7229	0.4940
	P8	0.5787	1.0769	–
	P9	0.1537	0.8800	1.8715
	H1	3.5392		
	H2	0.7454		
	H3	2.1402		
	H4	3.5406		
	H5	2.0589		
	H6	2.7509		
	H7	6.1721		
	H8	0.6712		
	H9	2.7146		
	H10	2.6164		
	H11	0.7193		
	H12	1.4663		
	H13	0.9191		
	H14	6.6107		
	H15	3.5822		
	H16	3.5711		
	H17	2.2981		
	H18	2.6966		
	H19	2.8326		

Abbreviations: *P* patient, *H* healthy controls. The percentages of EBV specific CTLs of healthy controls were detected at only one-time point during our study

### Comparison of HLA-A*1101/ EBV-LMP2-SSC-specific CTLs in patients with and without EBV-related diseases

Among the nine patients with HLA-A*1101/EBV-LMP2-SSC-specific CTL data, five showed no evidence of EBV reactivation, two had EBV-DNAemia, and two had EBV-related diseases; one patient who suffered from EBV-related gastroenteritis died.

EBV-DNA copies were not simultaneously detected in the peripheral blood of the patient with EBV-related gastroenteritis. We speculated that the detection of high EBV-DNA copies in blood was not always synchronous with EBV-related diseases. Therefore, we did not associate EBV-DNA copies with EBV-CTLs.

The mean percentages of HLA-A*1101/EBV-LMP2-SSC-specific CTLs at + 30, + 60, and + 90 days after haplo-HSCT in patients with and without EBV-related diseases were not significantly different (*p* = 0.500, 0.889, *and* 1.000, respectively).

### Comparison of HLA-A*0201/EBV-BMLF1-GLC-specific CTLs in patients with and without EBV-related diseases

Among the nine patients with HLA-A*0201/EBV-BMLF1-GLC-specific CTL data, three showed no evidence of EBV reactivation, three had EBV-DNAemia, and three had EBV-related diseases; two patients suffering from EBV-related diseases died. EBV-DNA copies were not simultaneously detected in the peripheral blood of the patient with EBV-related encephalitis. The characteristics of these nine patients are presented in Table 3.

**Table 3:** Characteristics of nine patients with HLA-A*0201

Patient No	EBV state	Percentages of EBV-CTLs at + 30 days	Percentages of EBV-CTLs at + 60 days	Percentages of EBV-CTLs at + 90 days	Time of CSA withdrawal	aGVHD	Result	Cause of death
1	No	0.1982	2.9100	1.0950		no	alive	
2	EBV-DNAemia	0.8337	1.9200	1.3644	20	Grade I	alive	
3	No	0.7296	3.0000	1.0119	19	Grade I	alive	
4	EBV-DNAemia	0.3252	3.1700	1.4483	–	Grade I	alive	
5	EBV-DNAemia	0.0179	1.9000	1.1990	16	Grade I	alive	
6	No	1.0790	4.2400	–	11	Grade I	dead	Aspergillus pneumonia
7	EBV-related disease	0.5619	0.6900	–	5	Grade I	dead	EBV encephalitis
8	EBV-related disease	0.2727	0.5300	–	–	no	alive	
9	EBV-related disease	0.9600	0.5500	–	58	Grade I	dead	EBV enteritis

Note: The patient who died at + 43 days was a 100% donor type by bone puncture and STR examination at + 30 days after transplantation. However, at + 33 days, this patient developed hyperthermia, pulmonary infection, and decreased blood cells and was found to have decreased chimerism to 23% of that of the donor recipient at + 37 days. Secondary graft failure was observed, which was thought to be related to the serious pulmonary fungal infection. We did not include this patient in this Table

**Fig. 5:**
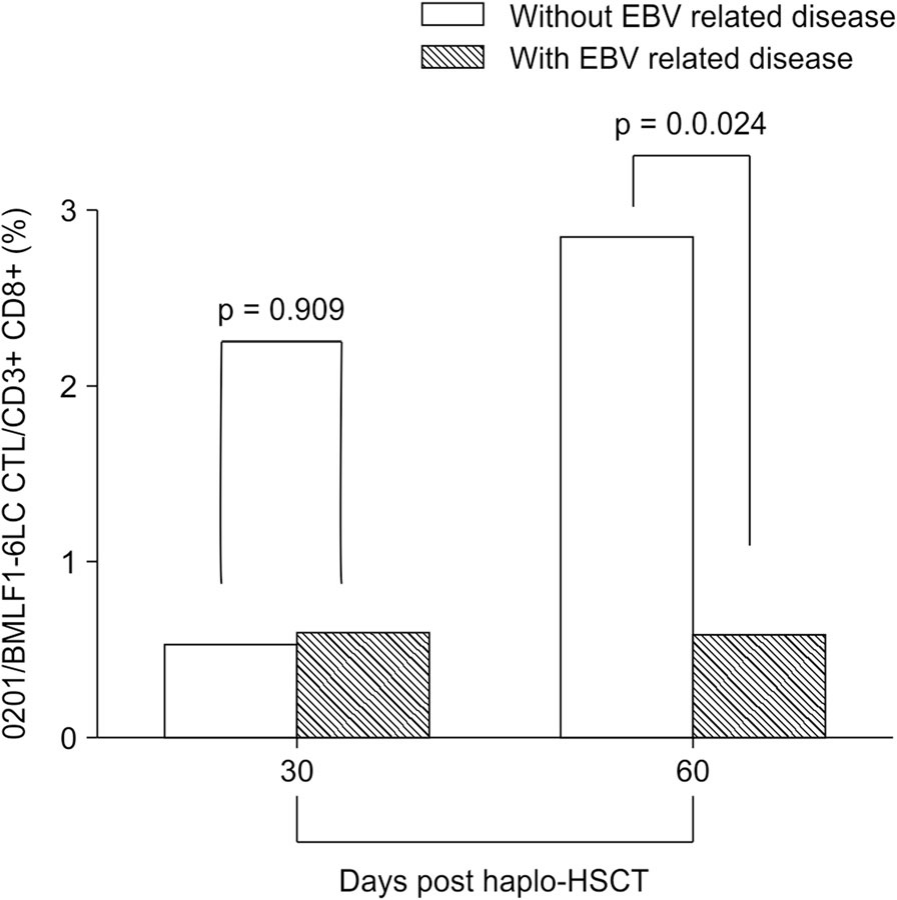
Comparison of HLA-A*0201/EBV-BMLF1-GLC-specific CD8^+^ T cells in patients with and without EBV-related diseases. The percentages of HLA-A*0201/BMLF1-GLC-specific CTLs at + 30 days after haplo-HSCT in patients with and without EBV-related diseases are shown. EBV-CTL percentage data were available for 5 patients at + 90 days in the HLA-A*0201 group, but none of these subjects suffered from EBV-related diseases. Thus, we did not analyze the difference of HLA-A*0201/EBV-BMLF1-GLC-specific CTLs at + 90 days

The mean percentages of HLA-A*0201/BMLF1-GLC-specific CTLs at + 30 days after haplo-HSCT in patients with and without EBV-related diseases were not significantly different (*p* = 0.909). However, there was a significant difference in the mean percentages of HLA-A*0201/BMLF1-GLC-specific CD8^+^ T cells at + 60 days between patients with and without EBV-related diseases (*p* = 0.024), with patients with EBV-related diseases showing a lower percentage than those without EBV-related disease (Fig. 5). Five patients had EBV-CTL percentage data at + 90 days in the HLA-A*0201 group, but none suffered from EBV-related diseases. Thus, we did not analyze the difference in HLA-A*0201/EBV-BMLF1-GLC-specific CTLs at + 90 days.

## Discussion

Multiple studies have suggested the importance of EBV-specific T cells in controlling EBV infections, particularly after HSCT [13, 14]. However, little is known about EBV-specific immune reconstitution after HSCT. The results of our current study provided insight into the early reconstitution of EBV-specific CD8^+^ T cells after haplo-HSCT with the RIC protocol including ATG. Our study of the dynamics of EBV-specific immune reconstitution in the early stage after haplo-HSCT will assist in determining the appropriate time point of EBV-specific immune therapy to prevent or treat EBV infections.

HLA-A*0201/BMLF1-GLC tetramers or pentamers can be used to detect EBV-CTLs recognizing the BMLF1 lytic cycle protein in healthy long-term EBV carriers, patients with infectious mononucleosis, and those who have undergone solid organ transplants or HSCT [15–18]. HLA-A*1101/LMP2-SSC tetramers are often used to detect EBV-CTLs against the LMP2 latent cycle protein in patients with nasopharyngeal carcinoma or lymphoma [19, 20], but are not frequently employed to screen transplant recipients. We detected EBV-CTLs using both GLC- and SSC-pentamers specific to the lytic cycle proteinBMLF1 and the latent cycle protein-LMP2, respectively, in all patients after haplo-HSCT and in healthy volunteers. The results indicate that the two pentamers were also useful for detecting EBV-CTLs in the peripheral blood of patients suffering from HSCT.

Samples from healthy adult volunteers produced negative and positive reactions in EBV-IgM and EBV-IgG serum arrays, respectively. Hence, the frequencies of EBV-CTLs may reflect the levels of EBV-CTLs in healthy long-term EBV carriers. In our study, the frequencies of EBV lytic antigen BMLF1-GLC and latent antigen LMP2-SSC associated with EBV-CTLs in healthy long-term EBV carriers were 2.222% ± 1.665 and 2.508% ± 1.766%, respectively. These results demonstrated that the frequencies of EBV-CTLs against the immediate early lytic cycle protein and latent cycle protein were comparable in healthy long-term EBV carriers. Another study also showed that EBV-specific CD8+ T cells against lytic protein epitopes are readily detectable in healthy EBV carriers [16]. However, the abundances of EBV-specific CD8^+^ T cells against lytic protein epitopes HLA*A2/GLC and HLA*B8/RAK were found to be higher than those against latent protein epitopes HLA*A2/SVR and HLA*B8/FLR, respectively [16]. The difference in the two studies indicates that larger sample sizes and a greater number of pentamers of different antigen epitopes are needed to detect statistical significance. Additionally, our results should be further verified using other methods, such as ELISPOT.

This study was a preliminary investigation of the dynamics of GLC-specific and SSC-specific EBV-CTL reconstitution after haplo-HSCT. Compared with healthy EBV carriers, GLC-specific EBV-CTLs appeared at + 30 days after haplo-HSCT, although at a significantly lower level, subsequently recovering to normal levels at + 60 days and decreasing slightly at + 90 days after haplo-HSCT. SSC-specific EBV-CTLs were detected in all nine patients at + 30, + 60, and + 90 days after haplo-HSCT; however, the frequencies were significantly lower than those in healthy EBV carriers, indicating that SSC-specific EBV-CTLs recognizing the latent protein, LMP2, did not recover to normal levels within 90 days after haplo-HSCT. Frequencies of HLA-A*1101/LMP2-SSC-specific CTLs at + 60 days were significantly lower than those of HLA-A*0201/EBV-BMLF1-GLC-specific CTLs. The frequencies of the two types of EBV-CTLs reached a balance at + 90 days after HSCT. The dynamics of EBV-specific immune reconstitution were similar to those of immune constitution observed in patients with infectious mononucleosis who exhibited abundant reactive EBV-CTLs against lytic cycle proteins, whereas EBV-CTLs against latent cycle peptides were detectable, but at low levels. From 3 to 8 months after acute infection, EBV-CTLs against lytic antigens gradually decreased, whereas those recognizing latent antigen began to increase until homeostasis between the two groups of EBV-CTLs was reached. Marshall et al. [21] reported similar findings in one post-transplant patient; at + 30 days after transplantation, the levels of EBV-CTLs against lytic and latent phase antigenic peptides were significantly lower in the patient. However, the EBV-CTLs against lytic or latent antigen were clearly increased at + 60 days, and the EBV-CTLs recognizing the lytic antigen were predominant. They also found that the EBV-specific immune response was recovered at + 30 days after HSCT.

These results regarding the kinetics of EBV-specific immune reconstitution can facilitate EBV-specific immunotherapy. Thus, we compared the levels of EBV-CTLs in patients with or without EBV-related diseases. Those with EBV-related diseases showed lower levels of GLC-specific EBV-CTLs at + 60 days after haplo-HSCT than those without EBV-related diseases. This indicated that patients with a lower response against the lytic protein BMLF1-GLC at + 60 days were at a higher risk of developing EBV-related diseases. Annels et al. [18] simultaneously analyzed viral loads and T cell reconstitution and arrived at a similar conclusion, demonstrating that the effectiveness of preemptive intervention may be limited in patients who lack EBV-specific T cell expansion during the initial phase of EBV reactivation. Our findings were also in accordance with the results reported by D’Aveni et al. [22], who monitored EBV-specific T cell quantification by Elispot assay at + 60, + 100, + 180, and + 360 days after HSCT. For cases in which EBV DNAemia occurred and cleared spontaneously, their ELISPOT results indicated the presence of more than > 1000 spot-forming cells per 10^6^ PBMCs.

This finding suggested that the low frequency of EBV-CTLs recognizing BMLF1-GLC at + 60 days after haplo-HSCT could be used as a marker for the occurrence of EBV-related diseases. Transfusion with EBV-CTLs within 60 days after haplo-HSCT may have prophylactic effects against EBV-related diseases. However, we analyzed a small number of cases which may have resulted in large deviations. Further studies with larger sample sizes are needed to verify our findings.

## Conclusion

In contrast to similar studies, the patients in our study were relatively homogeneous with regard to the type of donor, source of graft, conditioning, source and dose of ATG, and type of immunosuppressive prophylaxis. However, our study had a small number of patients, and thus the findings should be interpreted with caution. Overall, our data indicated that EBV-specific immune reconstitution after haplo-HSCT significantly affected the control of EBV-related diseases. Thus, transfusion with EBV-CTLs within 60 days after haplo-HSCT may have prophylactic effects against EBV-related diseases.

## Abbreviations

aGVHD: Acute graft-versus-host disease
APC: Allophycocyanin
ATG: Antithymocyte globulin
CSA: Cyclosporine A
CTLs: Cytotoxic (CD8^+^) T lymphocytes
EBV: Epstein Barr virus
FCS: Fetal calf serum
FITC: Fluorescein isothiocyanate
Haplo-HSCT: Haploidentical hematopoietic stem cell transplant
PBS: Phosphate-buffered saline
PCR: Polymerase chain reaction
PE: Phycoerythrin
PerCP: Peridinin chlorophyll protein
PTLDs: Post-transplant lymphoproliferative disorders
RIC: Reduced intensity conditioning

## References

[1] Raberahona M, Wackenheim C, Germi R, Carré M, Bulabois CE, Thiébaut A (2016). Dynamics of Epstein-Barr viral load after hematopoietic stem cell transplantation and effect of preemptive rituximab therapy. Transpl Infect Dis.

[2] García-Cadenas I, Castillo N, Martino R, Barba P, Esquirol A, Novelli S (2015). Impact of Epstein Barr virus-related complications after high-risk Allo-SCT in the era of pre-emptive rituximab. Bone Marrow Transplant.

[3] Gil L, Styczyński J, Komarnicki M (2012). Strategy of pre-emptive management of Epstein-Barr virus post-transplant lymphoproliferative disorder after stem cell transplantation: results of European transplant centers survey. Contemp Oncol (Pozn).

[4] Styczynski J, Gil L, Tridello G, Ljungman P, Donnelly JP, Van Der Velden W (2013). Response to rituximab-based therapy and risk factor analysis in Epstein Barr virus related lymphoproliferative disorder after hematopoietic stem cell transplant in children and adults: a study from the infectious diseases working Party of the European Group for blood and marrow transplantation. Clin Infect Dis.

[5] Uhlin M, Wikell H, Sundin M, Blennow O, Maeurer M, Ringden O (2014). Risk factors for Epstein-Barr virus-related post-transplant lymphoproliferative disease after allogeneic hematopoietic stem cell transplantation. Haematologica.

[6] Rouce RH, Louis CU, Heslop HE (2014). Epstein-Barr virus lymphoproliferative disease after hematopoietic stem cell transplant. Curr Opin Hematol.

[7] Cohen JM, Cooper N, Chakrabarti S, Thomson K, Samarasinghe S, Cubitt D, et al. EBV-related disease following haematopoietic stem cell transplantation with reduced intensity conditioning. Leuk Lymphoma. 2007;(2):256–69. 10.1080/10428190601059837 17325885

[8] Leen AM, Heslop HE, Brenner MK (2014). Antiviral T-cell therapy. Immunol Rev.

[9] Haque T, Wilkie GM, Jones MM, Higgins CD, Urquhart G, Wingate P (2007). Allogeneic cytotoxic T-cell therapy for EBV-positive posttransplantation lymphoproliferative disease: results of a phase 2 multicenter clinical trial. Blood.

[10] Leen AM, Bollard CM, Mendizabal AM, Shpall EJ, Szabolcs P, Antin JH (2013). Multicenter study of banked third-party virus-specific T cells to treat severe viral infections after hematopoietic stem cell transplantation. Blood.

[11] Dong L, Gao ZY, Chang LJ, Liang Y, Tan XY, Liu JH (2010). Adoptive transfer of cytomegalovirus/Epstein-Barr virus-specific immune effector cells for therapeutic and preventive/preemptive treatment of pediatric allogeneic cell transplant recipients. J Pediatr Hematol Oncol.

[12] Styczynski J, van der Velden W, Fox CP, Engelhard D, de la Camara R, Cordonnier C (2016). Sixth European conference on infections in leukemia, a joint venture of the infectious diseases working Party of the European Society of blood and marrow transplantation (EBMT-IDWP), the infectious diseases Group of the European Organization for research and treatment of Cancer (EORTC-IDG), the international immunocompromised host society (ICHS) and the European leukemia net (ELN). Management of Epstein-Barr Virus infections and post-transplant lymphoproliferative disorders in patients after allogeneic hematopoietic stem cell transplantation: sixth European conference on infections in leukemia (ECIL-6) guidelines. Haematologica.

[13] Sanz J, Andreu R (2014). Epstein-Barr virus-associated posttransplant lymphoproliferative disorder after allogeneic stem cell transplantation. Curr Opin Oncol.

[14] Li Q, Rane L, Poiret T, Zou J, Magalhaes I, Ahmed R (2016). Both high and low levels of cellular Epstein-Barr virus DNA in blood identify failure after hematologic stem cell transplantation in conjunction with acute GVHD and type of conditioning. Oncotarget.

[15] Hislop AD, Annels NE, Gudgeon NH, Leese AM, Rickinson AB (2002). Epitope-specific evolution of human CD8 (+) T cell responses from primary to persistent phases of Epstein-Barr virus infection. J Exp Med.

[16] Tan LC, Gudgeon N, Annels NE, Hansasuta P, O'Callaghan CA, Rowland-Jones S (1999). A re-evaluation of the frequency of CD8+ T cells specific for EBV in healthy virus carriers. J Immunol.

[17] Chakrabarti S, Milligan DW, Pillay D, Mackinnon S, Holder K, Kaur N (2003). Reconstitution of the Epstein-Barr virus-specific cytotoxic T-lymphocyte response following T-cell-depleted myeloablative and nonmyeloablative allogeneic stem cell transplantation. Blood.

[18] Annels NE, Kalpoe JS, Bredius RG, Claas EC, Kroes AC, Hislop AD (2006). Management of Epstein-Barr virus (EBV) reactivation after allogeneic stem cell transplantation by simultaneous analysis of EBV DNA load and EBV-specific T cell reconstitution. Clin Infect Dis.

[19] Straathof KC, Leen AM, Buza EL, Taylor G, Huls MH, Heslop HE (2005). Characterization of latent membrane protein 2 specificity in CTL lines from patients with EBV-positive nasopharyngeal carcinoma and lymphoma. J Immunol.

[20] Zheng Y, Parsonage G, Zhuang X, Machado LR, James CH, Salman A (2015). Human leukocyte antigen (HLA) a*1101-restricted Epstein-Barr virus-specific T-cell receptor gene transfer to target nasopharyngeal carcinoma. Cancer Immunol Res.

[21] Marshall NA, Howe JG, Formica R, Krause D, Wagner JE, Berliner N (2000). Rapid reconstitution of Epstein-Barr virus-specific T lymphocytes following allogeneic stem cell transplantation. Blood.

[22] D'Aveni M, Aïssi-Rothé L, Venard V, Salmon A, Falenga A, Decot V (2011). The clinical value of concomitant Epstein Barr virus (EBV)-DNA load and specific immune reconstitution monitoring after allogeneic hematopoietic stem cell transplantation. Transpl Immunol.

